# Influence of the Surgeon’s First Operation of the Day on Patient Outcomes

**DOI:** 10.1097/SLA.0000000000006666

**Published:** 2025-02-13

**Authors:** Filippo Dagnino, Stephanie Polazzi, Jean-Christophe Lifante, Tanujit Dey, Antoine Duclos, TopSurgeons Study Group, Jake Awtry

**Affiliations:** * Department of Urology, Humanitas Clinical and Research Hospital, Milan, Italy; † Department of Surgery, Center for Surgery and Public Health, Brigham and Women’s Hospital, Boston, MA; ‡ Research on Healthcare Performance RESHAPE, Inserm U1290, Université Claude Bernard Lyon 1, Lyon, France; § Department of Endocrine Surgery, Lyon Sud Hospital, Hospices Civil de Lyon, Lyon, France; ‖ Université Paris Cité and Université Sorbonne Paris Nord, Inserm, INRAE, Centre for Research in Epidemiology and Statistics (CRESS), METHODS Team, F-75004 Paris, France

**Keywords:** adverse event, complications, operative time, outcomes, workload

## Abstract

**Objective:**

To investigate whether the cumulative operative time spent by a surgeon operating on patients on the same day before starting a new procedure was associated with surgical outcomes.

**Background:**

The impact of daily operating room workload on a surgeon’s performance and patient outcomes is uncertain.

**Methods:**

All elective patients, operated by attending surgeons across 7 specialties in 4 French hospitals between November 1, 2020 and December 31, 2021, were included. Surgeons’ operative workload the same day before each operation was measured in minutes by cumulating incision-to-closure times for all their patients as the primary operator. Composite of adverse events within 30 days postsurgery, encompassed major surgical complications, unplanned reoperation, extended intensive care unit (ICU) stay, and patient death. Generalized linear mixed models estimated the association between each outcome and operative workload, considering the clustering of operations by surgeons, and adjusting for patient comorbidities, procedure complexity, and surgeon characteristics.

**Results:**

The cohort included 7979 elective surgeries performed by 44 surgeons. Composite adverse events rates were higher in the 0-minute group compared with those with a higher duration (20.7% vs 12.5%, *P* < 0.001), as were rates of major complications (19.3% vs 11.7%, *P* < 0.001), reoperations (6.5% vs 3.4%, *P* = 0.005), and ICU stay (3.7% vs 1.2%, *P* = 0.016). When the surgeon had already spent time operating on patients before the procedure, adjusted relative risks were lower for composite adverse events [adjusted relative risk: 0.85 (95% CI: 0.76 to 0.95)], major complications [0.86 (0.76 to 0.97)], reoperation [0.78 (0.63 to 0.97)], and ICU stay [0.69 (0.49 to 0.98)].

**Conclusions:**

First patient of the day may experience worse outcomes, prompting surgeons to warm up before starting surgery. Further research is needed to replicate these findings, as many surgeons may prioritize starting with the most complex and challenging cases, which inherently carry greater risks.

The performance of surgeons in the operating room may fluctuate over time and could be influenced by their daily workload. Workload encompasses both the volume of procedures performed and the overall time spent in surgery^1^ which depends on how their procedures are scheduled throughout the day.^2–4^ Daily case scheduling can have varying effects on surgical outcomes, with an inconsistent association for different specialties. Depending on the procedure type, this can result in either no effect or a fluctuating risk of adverse events for procedures performed later in the day.

The scheduling order of cases in the operating room can influence surgeons’ performance and lead to fluctuations in surgical outcomes.^2,5,6^ Fatigue accumulated by surgeons may contribute to poorer outcomes when they operate on patients later in the day.^7,8^ Conversely, surgeons may experience optimal performance after spending time operating earlier in the day, akin to a “warm-up” effect observed in sports.^9–11^ In surgical settings, a warm-up can involve preparatory activities or routines performed before any surgery, or gently starting with the first case of the day to ease into subsequent surgical procedures. Previous studies have shown promising effects of such warm-up processes on surgeons’ performance.^12,13^

The influence of daily workload and case scheduling in the operating room on a surgeon’s performance remains unclear regarding patient outcomes. Therefore, the purpose of this multispecialty cohort study was to determine the relationship between surgical outcomes and cumulative operative time spent by a surgeon on patients operated on earlier in the same day before starting a new procedure. Specifically, we aimed to investigate whether performing the first case of the day was associated with worse outcomes, thereby providing valuable insights into optimizing surgeons’ daily workflow patterns for patient safety.

## METHODS

### Study Design, Population and Exposure

We conducted a prospective observational cohort study across 14 surgical departments from 4 university hospitals in France specialized in digestive, orthopedics, gynecology, urology, cardiac, thoracic, and endocrine surgery. A cohort of 44 attending surgeons, each performing a minimum of 50 procedures per year, was formed. All elective surgical procedures they performed as the primary operator between November 1, 2020 and December 31, 2021, were considered in the final analysis. We excluded operations involving patients under 18 years old or those who refused to share personal data, as well as procedures for palliative care or organ donation, and those lacking operative timestamps. In addition, non-elective cases and surgeries performed at night were excluded from the analysis.

We investigated the relationship between patient outcomes and the cumulative operative time each surgeon spent in the operating room on the same day before starting each elective operation. Surgeon operative workload was quantified in minutes by summing the incision-to-closure durations for all elective and nonelective procedures where they served as the primary operators on the same day, excluding operations conducted during nighttime. The surgeon’s operative workload was primarily categorized as a dichotomous variable (ie, 0 vs more than 0 minutes), and secondarily into 4 categories close to the quartiles of the cumulative time spent operating on patients on the same day before each elective operation (ie, 0 minutes, more than 0 to 30 minutes, more than 30 to 90 minutes, and more than 90 minutes).

According to European General Data Protection Regulation No. 2016/679, this observational study was based on anonymous data and approved by the French Committee of Expertise in Research, Studies, and Evaluations in the Health domain (TPS_970330 CEREES), the French National Data Protection Authority (DR-2020-055 CNIL), and the European Research Council Executive Agency (801660 ERCEA). In addition, this study was deemed exempt from formal oversight by the Mass General Brigham Institutional Review Board (Protocol 2023P002266). Patients were informed that their health data might be reused for research purposes and that they had the opportunity to opt-out. Each surgeon in the cohort provided informed consent to participate in the research and for the use of their data.

### Data Sources and Outcomes

For each operation, data were prospectively collected from a homogeneous information system across the Lyon University hospitals, encompassing details regarding the operation, patient, surgeon, and operating room involved. Specifically, we gathered data on the patients’ sociodemographic characteristics (age, sex, social coverage, and median income), care provided during hospital stays, including details about the type of surgery performed (organ, surgical approach, and complexity), and the primary diagnosis linked to the operative indication.

We supplemented this information with data collected by clinical research assistants from the patients’ electronic health records to acquire details such as the scheduling of the operation (elective, semiurgent, and urgent), occurrences of intraoperative and postoperative adverse events, and the preoperative comorbidities of the patient (see Appendix Methods S1 for comorbidities list). We also extracted data from the anesthesia records to identify the type of anesthesia administered and the patients’ American Society of Anesthesiology (ASA) Physical Status Classification System.

In addition, for each operation, we used data from the operating room management software and the surgical procedure report to determine the timings of the patient’s entry into the operating room, the incision and closure of the wound, and the primary operating surgeon involved. Lastly, we gathered human resources data to determine the age and professional status of the operating surgeon.

For each operation conducted by a participating surgeon, information about the patient and surgeon was prospectively collected, including the minutes the surgeon had spent in the operating room before each elective surgery.

Inspired by the Clavien-Dindo classification, which considers surgical major adverse events in an objective and reproducible manner,^6^ the primary outcome was a composite measure encompassing major adverse events during surgery or within the 30-day postsurgery period. These events included major intraoperative or postoperative complications (see Supplemental Methods S1, Supplemental Digital Content 1, http://links.lww.com/SLA/F410 for detailed complications across various categories: general, infectious, hemorrhagic, parietal, cardiopulmonary, neurological, abdominopelvic, orthopedic, cervical, and functional), unplanned reoperation for complication arising from the initial surgery, extended stays postoperatively in the intensive care unit (ICU) for at least 2 nights or in the intermediate care unit for at least 5 nights due to organ failure, as well as intraoperative or postoperative mortality. For secondary outcomes, we considered each event separately.

### Statistical Analysis

Patient characteristics were presented using mean and SD for continuous variables, and frequencies with percentages for qualitative variables, whereas surgeon characteristics were presented using median and interquartile range for continuous variables. For each outcome, we compared the average team familiarity, based on the number of previous collaborations between surgeons, with respect to the occurrence of adverse events, and by considering the clustering of operations within surgeons.

We compared operation characteristics and patient outcomes based on the surgeon’s dichotomous cumulative operative workload, accounting for the clustering of operations within surgeons. We utilized generalized mixed logistic regression models with maximum likelihood estimation based on the Laplace approximation to evaluate the association between each patient outcome and the surgeon’s cumulative operative workload on the same day preceding each elective surgery, either as a dichotomous or 4-category exposure. In the final multivariable models, we included as fixed effects the log-transformed patient’s preoperative risk score, and the age and professional status (faculty position as associate/full professor or not) of the attending surgeon. The attending surgeon identity was also considered in the models as a random effect and possible interactions between the covariates were examined. Considering the tendency of adjusted odds ratios to overstate risk interpretation for outcomes with frequent occurrence, we computed adjusted relative risks (aRRs) and adjusted risk differences (aRDs) alongside their corresponding 95% CIs.^14^

It is important to note that the preoperative risk scores were created using outcome prediction models developed from an independent data set of patients operated by the same cohort of surgeons between January 1, 2022 and October 31, 2022 (Supplemental Methods S1, Supplemental Digital Content 1, http://links.lww.com/SLA/F410). Potential confounders considered for case-mix adjustment included patient age, sex, comorbidities, ASA score, socioeconomic status, patient comorbidities, surgical specialty, surgery indication and approach, procedure complexity and scheduling, and the type of anesthesia.

To assess the robustness of the study’s primary findings, we conducted a sensitivity analysis by stratifying the final models into 3 surgical specialty subgroups based on their varying baseline risk of adverse event occurrence: cardiac and thoracic surgery, digestive and endocrine surgery, and a combined group of orthopedic, urological, and gynecologic surgery.

We conducted additional sensitivity analyses to assess the robustness of the adjusted association between patient outcomes and the dichotomous measure of operative workload before initiating a new procedure. First, the analysis was stratified by quartiles of increasing surgical complexity. Second, we focused exclusively on procedures performed through an open surgical approach under general anesthesia. Third, we performed a stratified analysis based on procedure duration, categorizing them as “short” or “long” using the median duration of 67 minutes. Fourth, we analyzed the influence of procedure order within a surgeon’s daily schedule by grouping cases into 4 categories: first, second, third, and fourth or later, with the first case serving as the reference. Finally, we examined the effect of timing within the day, stratifying outcomes based on whether the surgical schedule began before or after noon.

All reported *P* values were 2-sided and we considered a value of <0.05 to be significant. Data manipulation and analyses were performed using R version 4.3.0 (R Foundation) and SAS software (version 9.4; SAS Institute Inc.).

## RESULTS

**FIGURE 1:**
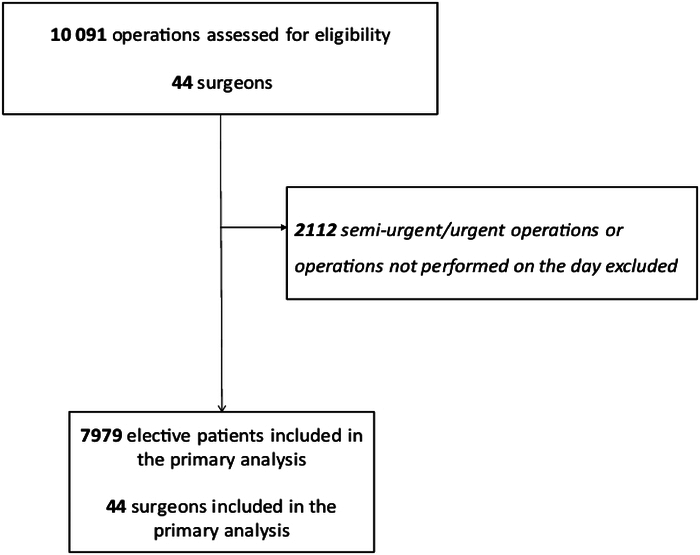
Population flowchart.

The final study population comprised 7979 elective operations performed by 44 surgeons (Fig. 1). Surgeons had a median age of 44 years, 77.3% were males, and 52.3% held full or associate professor positions (Table 1). Patients were predominantly females (57.6%) with a mean age of 55.3 years. On average, they had 2.3 comorbidities, and 23.6% had an ASA score of 3 or higher. The operations encompassed various specialties, including gynecologic, orthopedic, and urologic surgery (53.8%), digestive and endocrine surgery (32.8%), and cardiac and thoracic surgery (13.4%). Most were performed under general anesthesia (84.8%) and utilized an open approach (57.2%). Composite adverse events occurred in 1311 (16.4%) operations, while 1222 (15.3%) patients experienced major complications, 393 (4.9%) had an unplanned reoperation, 192 (2.4%) had a prolonged ICU stay, and 52 (0.7%) died. The average operative time from incision to wound closure was 97.5 minutes.

**TABLE 1:** Characteristics of Operations and Surgeons

Operations	Overall (n = 7979)
Patient sex, N (%)	
Male	3386 (42.4)
Female	4593 (57.6)
Patient age (yr), mean (SD)	55.3 (17.0)
Patient no. of comorbidities^*^, mean (SD)	2.3 (2.0)
Patient ASA score categories^†^, N (%)	
1	2306 (28.9)
2	3789 (47.5)
3	1822 (22.8)
4	60 (0.8)
5	2 (0.0)
Anesthesia technique (missing = 8), N (%)	
General	6758 (84.8)
Regional	1021 (12.8)
Local	192 (2.4)
Surgical specialty, N (%)	
Cardiac surgery	694 (8.7)
Endocrine surgery	751 (9.4)
Digestive surgery	1866 (23.4)
Gynecology	1970 (24.7)
Orthopedics	1506 (18.9)
Thoracic surgery	379 (4.7)
Urology	813 (10.2)
Surgical approach (missing = 53), N (%)	
Open	4532 (57.2)
Robot	383 (4.8)
Videoscopic	2094 (26.4)
Endoscopic	917 (11.6)
Operative time between incision and wound closure (min), mean (SD)	97.5 (98.3)
Surgeons	Overall (N = 44)
Men	34 (77.3)
Women	10 (22.7)
Surgeon age (yr), median (IQR)	44.0 (37.7 to 51.2)
Faculty status, N (%)	
Associate/full professor	23 (52.3)
Non-professor	21 (47.7)

^*^ Comorbidities including critical condition, current pregnancy, obesity, malnourishment, tobacco addiction, alcohol addiction, other addiction, open wound, surgical site infection, sepsis, endocarditis, cancer, neoadjuvant treatment, immune deficiency, coagulopathy, anticoagulant treatment, anti-aggregation treatment, blood transfusion, coma, limb paralysis, other neurological disorder, confusion, dementia, depression, cardiovascular disease, neurovascular disease, peripheral arterial disease, cardiac arrhythmia, chronic heart failure, hypertension, diabetes, dyslipidemia, high pulmonary artery systolic pressure, chronic renal failure, acute renal failure, chronic respiratory failure, chronic obstructive pulmonary disease, liver disease, rheumatic pathology, and hypoparathyroidism.

^†^ American Society of Anesthesiologists’ score.

IQR indicates interquartile range.

**FIGURE 2:**
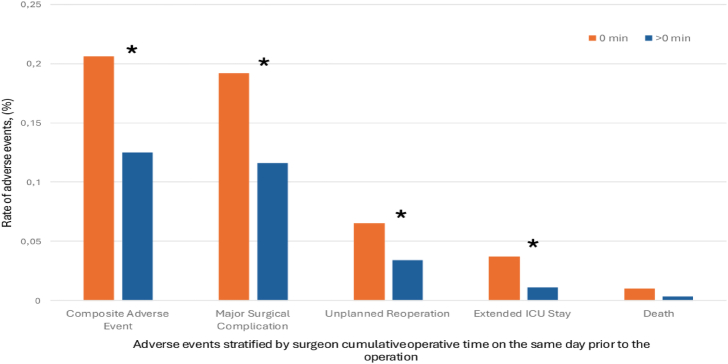
Unadjusted comparison of surgical outcomes based on surgeon cumulative operative time on the same day before the operation.

Table 2 compares the characteristics of operations and surgeons based on the cumulative operative time of the surgeon before every surgery. As shown in Figure 2, the unadjusted adverse event rates were significantly higher for patients undergoing surgery scheduled as the first case of the day (0-minute group) versus those scheduled later in the day (>0-minute group). Specifically, the rates were 20.7% versus 12.5% (*P* < 0.001) for composite adverse events, 19.3% versus 11.7% (*P* < 0.001) for major surgical complications, 6.5% versus 3.4% (*P* = 0.005) for unplanned reoperations, 3.7% versus 1.2% (*P* = 0.016) for extended ICU stays, and 1.0% versus 0.3% (*P* = 0.235) for inpatient deaths. Details by exposure categories (ie, 0 minutes, 0 to 30, 30 to 90, and >90 minutes) are also displayed in Supplemental eFigure 1 (Supplemental Digital Content eFig. 1, http://links.lww.com/SLA/F410).

**TABLE 2:** Comparison of Operations and Surgeon Characteristics Based on Surgeon Cumulative Operative Time on the Same Day Before the Operation

	0 min; 3838 (48.1)	>0 min; 4141 (51.9)	*P* ^‡^
Patient sex, N (%)			0.063
Male	1722/3386 (50.9)	1664/3386 (49.1)	—
Female	2116/4593 (46.1)	2477/4593 (53.9)	—
Patient age (yr), mean (SD)	55.9 (16.7)	54.9 (17.2)	0.196
Patient no. of comorbidities^*^, mean (SD)	2.4 (2.1)	2.1 (2.0)	<0.001
Patient ASA score^†^, mean (SD)	2.0 (0.8)	1.9 (0.7)	<0.001
Anesthesia technique (missing = 8), N (%)			<0.001
General	3496/6758 (51.7)	3262/6758 (48.3)	—
Regional	292/1021 (28.6)	729/1021 (71.4)	—
Local	46/192 (24.0)	146/192 (76.0)	—
Surgical approach (missing = 53), N (%)			<0.001
Open	2148/4532 (47.4)	2384/4532 (52.6)	—
Robot	240/383 (62.7)	143/383 (37.3)	—
Videoscopic	1078/2094 (51.5)	1016/2094 (48.5)	—
Endoscopic	347/917 (37.8)	570/917 (62.2)	—
Operative time between incision and wound closure (min), mean (SD)	124.6 (119.1)	72.3 (64.6)	<0.001
Surgeon sex, N (%)			0.070
Male	3005/5885 (51.1)	2880/5885 (48.9)	—
Female	833/2094 (39.7)	1261/2094 (60.2)	—
Surgeon age (yr), mean (SD)	45.5 (8.5)	45.3 (8.5)	0.872
Surgeon faculty status, N (%)			0.530
Associate/full professor	2203/4462 (49.4)	2259/4462 (50.6)	—
Non-professor	1635/3517 (46.5)	1882/3517 (53.5)	—
Surgical specialty grouped, N (%)			0.006
Cardiac-thoracic surgery	685/1073 (63.8)	388/1073 (36.2)	—
Digestive-endocrine surgery	1362/2617 (52.0)	1255/2617 (48.0)	—
Orthopedics-urology-gynecology	1791/4289 (41.8)	2498/4289 (58.2)	—

^*^ Comorbidities including critical condition, current pregnancy, obesity, malnourishment, tobacco addiction, alcohol addiction, other addiction, open wound, surgical site infection, sepsis, endocarditis, cancer, neoadjuvant treatment, immune deficiency, coagulopathy, anticoagulant treatment, anti-aggregating treatment, blood transfusion, coma, limb paralysis, other neurological disorder, confusion, dementia, depression, cardiovascular disease, neurovascular disease, peripheral arterial disease, cardiac arrhythmia, chronic heart failure, hypertension, diabetes, dyslipidemia, high pulmonary artery systolic pressure, chronic renal failure, acute renal failure, chronic respiratory failure, chronic obstructive pulmonary disease, liver disease, rheumatic pathology, and hypoparathyroidism.

^†^ The ASA score is reported as a continuous variable.

^‡^ Pearson χ^2^ test; Wilcoxon rank sum test; Fisher exact test.

Table 3 and Supplemental eFigure 2 (Supplemental Digital Content eFig. 2, http://links.lww.com/SLA/F410), as well as Supplemental eTable 1 (Supplemental Digital Content eTable 1, http://links.lww.com/SLA/F410) and Supplemental eTable 2 (Supplemental Digital Content eTable 2, http://links.lww.com/SLA/F410), provide adjusted risk estimates from multivariable models. Compared with the first operation of the day, the risk was lower when the surgeon had already spent time operating on patients on that same day for composite adverse events [aRR: 0.85; (95% CI: 0.76 to 0.95); aRD: −3.1%; (95% CI: −5.0% to −1.0%)], major surgical complications [aRR: 0.86; (95% CI: 0.76 to 0.97); aRD: −2.7%; (95% CI: −4.6% to −0.6%)], unplanned reoperation [aRR: 0.78; (95% CI: 0.63 to 0.97); aRD: −1.4%; (95% CI: −2.4% to −0.2%)], and extended ICU stay [aRR: 0.69; (95% CI: 0.49 to 0.98); aRD: −1.2%; (95% CI: −1.9% to −0.1%)]. Overall, the risk tended to be lower when surgeons had spent more than 30 minutes operating on patients on the same day for composite adverse events [aRR: 0.81; (95% CI: 0.68 to 0.95); aRD: −4.0%; (95% CI: −6.6% to −1.0%)] and major complications [aRR: 0.84; (95% CI: 0.71 to 0.99); aRD, −3.1%; (95% CI: −5.6% to −0.2%)], and when surgeons had spent more than 90 minutes for operations [aRR, 0.76; (95% CI: 0.59 to 1.00); aRD: −1.5%; (95% CI: −2.7% to 0.1%)] and ICU stays [aRR: 0.63; (95% CI: 0.41 to 0.95); aRD: −1.4%; (95% CI: −2.2% to −0.2%)].

**TABLE 3:** aRR^*^ and aRD for Each Outcome Based on Surgeon's Cumulative Operative Time on the Same Day Before the Operation

	Composite adverse events		Major surgical complications		Unplanned reoperation		Extended stay in ICU		Death	
	aRR (95% CI)	aRD (%) (95% CI)	aRR (95% CI)	aRD (%) (95% CI)	aRR (95% CI)	aRD (%) (95% CI)	aRR (95% CI)	aRD (%) (95% CI)	aRR (95% CI)	aRD (%) (95% CI)
Cumulative operative time the same day until operation (min; dichotomized)										
0	Reference	—	Reference	—	Reference	—	Reference	—	Reference	—
>0	0.85 (0.76–0.95)	−3.1 (−5.0 to −1.0)	0.86 (0.76 to 0.97)	−2.7 (−4.6 to −0.6)	0.78 (0.63 to 0.97)	−1.4 (−2.4 to −0.2)	0.69 (0.49 to 0.98)	−1.2 (−1.9 to −0.1)	0.65 (0.33 to 1.28)	−0.3 (−0.7 to 0.3)
Cumulative operative time the same day until operation (min; 4 categories)										
>0 to 30	0.90 (0.73 to 1.12)	−2.0 (−5.6 to 2.4)	0.89 (0.71 to 1.12)	−2.1 (−5.6 to 2.3)	0.93 (0.62 to 1.42)	−0.4 (−2.5 to 2.7)	1.00 (0.46 to 2.14)	0.0 (−2.0 to 4.2)	—	—
>30 to 90	0.81 (0.68 to 0.95)	−4.0 (−6.6 to −1.0)	0.84 (0.71 to 0.99)	−3.1 (−5.6 to −0.2)	0.75 (0.54 to 1.05)	−1.6 (−3.0 to 0.3)	0.75 (0.40 to 1.37)	−0.9 (−2.2 to 1.4)	—	—
>90	0.86 (0.75 to 0.98)	−2.9 (−5.2 to −0.5)	0.87 (0.75 to 1.00)	−2.6 (−4.8 to 0.1)	0.76 (0.59 to 1.00)	−1.5 (−2.7 to 0.1)	0.63 (0.41 to 0.95)	−1.4 (−2.2 to −0.2)	—	—

^*^ Adjusted rate ratios and corresponding risk reduction were adjusted for the patient preoperative risk, surgeon’s age, and surgeon’s faculty status.

Compared with the main analysis, the sensitivity analysis by surgical specialty subgroups revealed robust findings indicating a higher risk associated with the first operation of the day performed by the surgeon for digestive and endocrine surgery, as well as a nonsignificant trend for the combined group of orthopedic, urological, and gynecologic surgery. However, those findings were not consistently observed for cardiac and thoracic surgery (Supplemental Digital Content eFig. 3, http://links.lww.com/SLA/F410).

When stratifying the analysis by surgical complexity, we consistently observed either statistically significant differences or trends indicating a higher risk of adverse events for the first case of the daily schedule across all quartiles (Supplemental Digital Content eTable 3, http://links.lww.com/SLA/F410). The results remained consistent with our main findings when focusing specifically on open procedures performed under general anesthesia (Supplemental Digital Content eTable 4, http://links.lww.com/SLA/F410). Similarly, when the analysis was stratified by procedure duration, we observed trends toward a higher risk of adverse events for the first case of the day, with this association being more pronounced for “short” procedures compared with “long” procedures (Supplemental Digital Content eTable 5, http://links.lww.com/SLA/F410). Further analysis of procedure order revealed a higher risk of adverse events for the first case of the day, followed by a gradual decline in risk estimates as the day progressed (Supplemental Digital Content eTable 6, http://links.lww.com/SLA/F410). Notably, the elevated risk for the first procedure was primarily observed when the individual surgeon’s schedule began before noon, whereas no such association was found for schedules starting after noon (Supplemental Digital Content eTable 7, http://links.lww.com/SLA/F410).

## DISCUSSION

In this prospective multispecialty cohort study, we investigated the relationship between the cumulative operating time of surgeons on the same day before starting a new procedure and the occurrence of surgical adverse events. Our findings indicate that procedures conducted as the first case of the day had higher rates of adverse events compared with those conducted later in the day. Significant differences were observed between these two groups across various patient outcomes, including composite adverse events, major surgical complications, unplanned reoperations, and extended ICU stays. Furthermore, patients operated on by surgeons who had already spent more than 30 minutes in prior operations before their procedure tended to experience better outcomes. When we stratified the analysis by surgical specialty subgroups, we observed that the strongest association between conducting the initial operation of the day and increased risk of adverse events was found in digestive and endocrine surgeries. In orthopedic, urological, and gynecologic surgeries, we also observed a trend towards increased risk, although it did not reach statistical significance. In contrast, no apparent association was identified in cardiac and thoracic surgeries. Sensitivity analyses strongly supported the main findings. Stratification by surgical complexity consistently showed a higher risk of adverse events for the first procedure of the day compared with subsequent cases. In a subsample of cases involving an open approach under general anesthesia, the first procedure of the day remained the riskiest. Similarly, when accounting for procedure duration, the first case in the schedule continued to exhibit a higher risk of adverse events, particularly for shorter procedures. Moreover, an analysis of the case order revealed a declining risk of adverse events as the schedule progressed, suggesting a potential dose-response relationship throughout the surgical schedule. Finally, we found that the increased risk of adverse events was primarily associated with the first case of the day when the surgeon’s schedule began in the morning, but not in the afternoon.

Available evidence regarding the influence of surgeons’ prior workload on subsequent procedures on the same day remains limited and inconclusive. In orthopedics, the impact of performing arthroplasty later in the daily schedule compared with the first case is still uncertain.^15,16^ In cataract surgery, the first case of the day^17^ was longer than subsequent cases. In urology, later cases of the day in robotic prostatectomy^18^ were associated with decreased operative time, while performing several complex procedures consecutively did not lead to worse outcomes.^19^ In interventional gastroenterology, polyp detection rates decreased as the gastroenterologist’s daily schedule progressed.^20^ In spine surgery, decompressive procedures performed later in the day carry a higher risk for postoperative infection, but not fusion procedures.^21^ In cardiac surgery, non-first procedures were associated with poorer outcomes in technically challenging surgical procedures like off-pump coronary bypass graft surgery but not for on-pump procedures.^22^ Overall, previous studies have revealed inconclusive findings regarding surgeons’ performance for procedures performed later in the day. Depending on the surgical procedure, some studies have reported shorter operative times, whereas others have found either unchanged or worse outcomes for later operations.^15–18^ In contrast, our multispecialty study demonstrated better outcomes for procedures conducted after the first case of the day. This discrepancy may be due to the fact that most studies examining the impact of surgeons’ prior workload on subsequent procedures do not fully account for their entire daily surgical activity.^23^ Our research included all types of procedures performed by a surgeon throughout their daily schedule, providing a more comprehensive perspective on their overall activity. In addition, our study encompassed various surgical specialties, whereas previous studies have been limited to specific procedures.

Confidence in our findings is bolstered by several elements. The prospective design of our study enabled accurate monitoring of surgeons’ cumulative operative time on the same day before each procedure and the subsequent occurrence of associated adverse events. We also ensured diversity by including surgeons from multiple specialties and departments. Comprehensive data were meticulously gathered from this multi-institutional cohort of surgeons and rigorously evaluated through systematic scrutiny of patient health records by research assistants. To enhance validity, these records were cross-referenced with other sources, mitigating potential biases and ensuring data integrity compared to relying on the secondary use of large hospital databases. Furthermore, we controlled for potential confounding factors, such as operation and surgeon characteristics, and validated our predictive models of surgical outcomes using an independent data set to assess preoperative patient risk. Ultimately, our analysis produced robust results across a range of outcome measures and across most surgical specialties.

Our findings indicate that the first procedure of the day is the riskiest, suggesting the idea that surgeons may benefit from a “warm-up” routine before starting a long day in the operating room to achieve optimal psychomotor and cognitive performance. Preoperative warm-up exercises with simple surgical tasks have the potential to increase surgical proficiency.^24^ Simulating basic surgical techniques positively impact trainees’ laparoscopic performance, and procedure-specific preoperative warm-ups can lead to better surgical outcomes.^13,25^ A warm-up routine may be particularly beneficial for specialties characterized by daily schedules that include multiple elective operations of short to intermediate duration, such as minor digestive and endocrine procedures, orthopedic surgeries, and endoscopic procedures in urology and gynecology. Conversely, in specialties involving longer procedures with intrinsically high-risk patients, such as cardiac and thoracic surgeries, the potential benefits on patient outcomes may be limited. Another potential implication of our findings is the implementation of a dedicated warm-up room in the operating theater. This shared space would be used by surgeons to perform physical and simulation warm-up exercises before starting their daily surgical schedule.

### Limitations

It is important to acknowledge several limitations inherent in our study. The heterogeneous nature of the surgical population posed challenges in adjusting for case-mix, and there may be residual factors that could potentially confound our results. Despite our efforts to account for a wide range of patient, operation, and surgeon-specific variables in our adjustment process, there remains the possibility that other unknown or unmeasured factors could influence the relationship between a surgeon’s daily workload and patient outcomes. Given the observational design of this study, we could not ascertain whether the order of cases within the daily schedule was genuinely randomized. In practice, many surgeons may start their day with the more complex and high-risk procedures, often performed as the initial cases, which naturally carry a greater likelihood of adverse events. As a result, some surgeons in our cohort may have prioritized these challenging cases earlier in the day, potentially limiting our ability to fully account for this aspect of case complexity. Therefore, further research is needed to validate these findings. Furthermore, other potential confounders, such as factors related to the anesthesia team and postoperative care, were not included in our analyses, as they were not captured in the available data. Lastly, the generalizability of our findings is constrained by the specific context of our study. Therefore, caution should be exercised when applying our results to different health care settings or populations outside of this scope.

## CONCLUSIONS

Within the daily surgical schedule of elective cases, the first procedure was associated with a higher risk of worse outcomes. These findings suggest that surgeons might benefit from incorporating a warm-up routine before starting their day in the operating room.

## Supplementary Material
