# Supplementary material for: Influence of the Surgeon’s First Operation of the Day on Patient Outcomes
Source: Ann Surg. 2025 Feb 13;284(1):43–50. doi: 10.1097/SLA.0000000000006666 (PMC13258112; doi:10.1097/SLA.0000000000006666)
Supplement: Supplementary file 1 [file sla-284-043-s001.docx]

**SUPPLEMENT 1**

- **eTable 1. Adjusted Odds Ratios (aOR) for each outcome based on Surgeon Cumulative Operative Time on the Same Day Prior to the Operation (binary exposure)**
- **eTable 2 Adjusted Odds Ratios (aOR) for each outcome based on Surgeon Cumulative Operative Time on the Same Day Prior to the Operation (four categories)**
- **eTable 3 Adjusted Odds Ratios (aOR) for each outcome based on Surgeon Cumulative Operative Time on the Same Day Prior to the Operation by Quartiles of Surgical Complexity**
- **eTable 4. Adjusted Odds Ratios (aOR) for each outcome based on Surgeon Cumulative Operative Time on the Same Day Prior to the Operation (binary exposure) in Surgical Cases Performed with Open Approach Under General Anesthesia**
- **eTable 5 Adjusted Odds Ratios (aOR) for each outcome based on Surgeon Cumulative Operative Time on the Same Day Prior to the Operation by Procedure Duration (median time of 67 minutes as cut-off)**
- **eTable 6 Adjusted Odds Ratios (aOR) for each outcome based on Order of Procedures Throughout the Daily Schedule**
- **eTable 7. Adjusted Odds Ratios (aOR) for each outcome based on Surgeon Cumulative Operative Time on the Same Day Prior to the Operation (binary exposure) in Surgical Cases Performed Before or After Noon**
- **eFigure 1. Unadjusted Comparison of Surgical Outcomes based on Surgeon Cumulative Operative Time on the Same Day Prior to the Operation grouped in four categories**
- **eFigure 2. Forest Plot overall elective population**
- **eFigure 3. Forest Plot elective population stratified by specialty groups**
- **Methods S1. Patient’s preoperative risk score development**

**eTable 1. Adjusted Odds Ratios (aOR) for each outcome based on Surgeon Cumulative Operative Time on the Same Day Prior to the Operation (binary exposure)**

|  | Composite Adverse Events | | Major Surgical Complications | | Unplanned Reoperation | | Extended Stay in Intensive Care Unit | | Death | |
| --- | --- | --- | --- | --- | --- | --- | --- | --- | --- | --- |
|  | **aOR**  **(95% CI)** | ***P*** | **aOR**  **(95% CI)** | ***P*** | **aOR**  **(95% CI)** | ***P*** | **aOR**  **(95% CI)** | ***P*** | **aOR**  **(95% CI)** | ***P*** |
| Cumulative Operative Time spent the Same Day until Operation | | | | | | | | | | |
| 0 min | Ref. | - | Ref. | - | Ref. | - | Ref. | - | Ref. | - |
| >0 min | 0.82  (0.71-0.94) | 0.004 | 0.83  (0.72-0.96) | 0.012 | 0.77  (0.61-0.97) | 0.028 | 0.68  (0.48-0.98) | 0.039 | 0.65  (0.33-1.28) | 0.211 |
| Risk score |  | | | | | | | | | |
| By one unit increase of Risk score logarithmic transformation | 2.01  (1.86-2.17) | 0.001 | 2.12  (1.95-2.30) | 0.001 | 1.57  (1.42-1.73) | 0.001 | 2.79  (2.37-3.28) | 0.001 | 1.86  (1.53-2.25) | 0.001 |
| Surgeon Faculty Status | | | | | | | | | | |
| Non-Professor | Ref. | - | Ref. | - | Ref. | - | Ref. | - | Ref. | - |
| Associate/Full-Professor | 0.96  (0.68-1.33) | 0.788 | 0.97  (0.69-1.36) | 0.863 | 1.14  (0.75-1.72) | 0.533 | 0.64  (0.33-1.25) | 0.190 | 0.43  (0.12-1.54) | 0.195 |
| Surgeons Age | | | | | | | | | | |
| By one year increase | 1.00  (0.98-1.02) | 0.690 | 1.01  (0.99-1.03) | 0.565 | 1.01  (0.99-1.03) | 0.458 | - | - | 1.03  (0.96-1.10) | 0.397 |

**eTable 2 Adjusted Odds Ratios (aOR) for each outcome based on Surgeon Cumulative Operative Time on the Same Day Prior to the Operation (four categories)**

|  | Composite Adverse Events | | Major Surgical Complications | | Unplanned Reoperation | | Extended Stay in Intensive Care Unit | | Death * | |
| --- | --- | --- | --- | --- | --- | --- | --- | --- | --- | --- |
|  | **aOR**  **(95% CI)** | ***P*** | **aOR**  **(95% CI)** | ***P*** | **aOR**  **(95% CI)** | ***P*** | **aOR**  **(95% CI)** | ***P*** | **aOR**  **(95% CI)** | ***P*** |
| Cumulative Operative Time spent the Same Day until Operation | | | | | | | | | | |
| 0 min | Ref. | - | Ref. | - | Ref. | - | Ref. | - | - | - |
| >0-30 min | 0.88  (0.68-1.15) | 0.363 | 0.87  (0.66-1.15) | 0.339 | 0.93  (0.60-1.46) | 0.767 | 1.00  (0.45-2.24) | 0.998 | - | - |
| >30-90 min | 0.77  (0.63-0.94) | 0.010 | 0.81  (0.66-0.99) | 0.039 | 0.74  (0.52-1.05) | 0.092 | 0.74  (0.39-1.39) | 0.347 | - | - |
| >90 min | 0.83  (0.70-0.97) | 0.023 | 0.84  (0.71-1.00) | 0.045 | 0.75  (0.57-1.00) | 0.046 | 0.62  (0.40-0.95) | 0.030 | - | - |
| Risk score |  | | | | | | | | | |
| By one unit increase of Risk score logarithmic transformation | 2.01  (1.86-2.17) | < 0.001 | 2.12  (1.95-2.30) | < 0.001 | 1.57  (1.42-1.73) | < 0.001 | 2.80  (2.37-3.30) | < 0.001 | - | - |
| Surgeon Faculty Status |  | | | | | | | | | |
| Non-Professor | Ref. | - | Ref. | - | Ref. | - | - | - | - | - |
| Associate/Full-Professor | 0.93  (0.68-1.28) | 0.666 | 0.97  (0.69-1.36) | 0.862 | 1.13  (0.75-1.71) | 0.557 | - | - | - | - |
| Surgeons Age |  | | | | | | | | | |
| By one year increase | 1.00  (0.99-1.02) | 0.607 | 1.01  (0.99-1.03) | 0.569 | 1.01  (0.99-1.03) | 0.468 | 1.00  (0.96-1.04) | 0.935 | - | - |

* Results regarding inpatient death are not provided due to the non-convergence of the model because of the insufficient number of events

**eTable 3 Adjusted Odds Ratios (aOR) for each outcome based on Surgeon Cumulative Operative Time on the Same Day Prior to the Operation by Quartiles of Surgical Complexity** ***^f^***

|  | Composite Adverse Events | | Major Surgical Complications | | Unplanned Reoperation | | Extended Stay in Intensive Care Unit * | | Death * | |
| --- | --- | --- | --- | --- | --- | --- | --- | --- | --- | --- |
|  | **aOR**  **(95% CI)** | ***P*** | **aOR**  **(95% CI)** | ***P*** | **aOR**  **(95% CI)** | ***P*** | **aOR**  **(95% CI)** | ***P*** | **aOR**  **(95% CI)** | ***P*** |
| Cumulative Operative Time spent the Same Day until Operation (by surgical complexity quartiles) | | | | | | | | | | |
| 0 min | Ref. | - | Ref. | - | Ref. | - | - | - | - | - |
| >0 min (1° quartile) | 0.75  (0.50-1.13) | 0.16 | 0.85  (0.53-1.36) | 0.50 | 1.29  (0.55-3.02) | 0.55 | - | - | - | - |
| >0 min (2° quartile) | 0.92  (0.66-1.29) | 0.65 | 0.86  (0.61-1.22) | 0.41 | 0.65  (0.37-1.11) | 0.11 | - | - | - | - |
| >0 min (3° quartile) | 0.72  (0.57-0.93) | 0.01 | 0.73  (0.56-0.94) | 0.01 | 0.64  (0.41-0.98) | 0.04 | - | - | - | - |
| >0 min (4° quartile) | 0.83  (0.67-1.03) | 0.09 | 0.86  (0.70-1.08) | 0.19 | 0.82  (0.58-1.17) | 0.27 | - | - | - | - |

*^f^* Odds ratios were adjusted for the patient preoperative risk, surgeon’s age, and surgeon’s faculty status

* Results regarding inpatient death are not provided due to the non-convergence of the model because of the insufficient number of events

**eTable 4. Adjusted Odds Ratios (aOR) for each outcome based on Surgeon Cumulative Operative Time on the Same Day Prior to the Operation (binary exposure) in Surgical Cases Performed with Open Approach Under General Anesthesia *^f^***

|  | Composite Adverse Events | | Major Surgical Complications | | Unplanned Reoperation | | Extended Stay in Intensive Care Unit | | Death | |
| --- | --- | --- | --- | --- | --- | --- | --- | --- | --- | --- |
|  | **aOR**  **(95% CI)** | ***P*** | **aOR**  **(95% CI)** | ***P*** | **aOR**  **(95% CI)** | ***P*** | **aOR**  **(95% CI)** | ***P*** | **aOR**  **(95% CI)** | ***P*** |
| Cumulative Operative Time spent the Same Day until Operation in Open Procedures Under General Anesthesia | | | | | | | | | | |
| 0 min | Ref. | - | Ref. | - | Ref. | - | Ref. | - | Ref. | - |
| >0 min | 0.81  (0.68-0.97) | 0.02 | 0.84  (0.70-1.01) | 0.06 | 0.76  (0.57-1.01) | 0.05 | 0.72  (0.49-1.08) | 0.12 | 0.65  (0.30-1.42) | 0.3 |

*^f^* Odds ratios were adjusted for the patient preoperative risk, surgeon’s age, and surgeon’s faculty status

**eTable 5 Adjusted Odds Ratios (aOR) for each outcome based on Surgeon Cumulative Operative Time on the Same Day Prior to the Operation by Procedure Duration (median time of 67 minutes as cut-off) *^f^***

|  | Composite Adverse Events | | Major Surgical Complications | | Unplanned Reoperation | | Extended Stay in Intensive Care Unit | | Death | |
| --- | --- | --- | --- | --- | --- | --- | --- | --- | --- | --- |
|  | **aOR**  **(95% CI)** | ***P*** | **aOR**  **(95% CI)** | ***P*** | **aOR**  **(95% CI)** | ***P*** | **aOR**  **(95% CI)** | ***P*** | **aOR**  **(95% CI)** | ***P*** |
| Cumulative Operative Time spent the Same Day until Operation by Procedure Duration | | | | | | | | | | |
| 0 min | Ref. | - | Ref. | - | Ref. | - | Ref. | - | Ref. | - |
| >0 min (short surgery) | 0.83  (0.64-1.07) | 0.2 | 0.95  (0.72-1.24) | 0.7 | 0.57  (0.37-0.89) | 0.01 | 0.54  (0.18-1.58) | 0.3 | 0.41  (0.06-2.63) | 0.3 |
| >0 min (long surgery) | 0.90  (0.76-1.06) | 0.2 | 0.89  (0.75-1.05) | 0.2 | 0.93  (0.71-1.23) | 0.6 | 0.77  (0.53-1.13) | 0.2 | 0.76  (0.36-1.60) | 0.5 |

*^f^* Odds ratios were adjusted for the patient preoperative risk, surgeon’s age, and surgeon’s faculty status

**eTable 6 Adjusted Odds Ratios (aOR) for each outcome based on Order of Procedures Throughout the Daily Schedule *^f^***

|  | Composite Adverse Events | | Major Surgical Complications | | Unplanned Reoperation | | Extended Stay in Intensive Care Unit | | Death * | |
| --- | --- | --- | --- | --- | --- | --- | --- | --- | --- | --- |
|  | **aOR**  **(95% CI)** | ***P*** | **aOR**  **(95% CI)** | ***P*** | **aOR**  **(95% CI)** | ***P*** | **aOR**  **(95% CI)** | ***P*** | **aOR**  **(95% CI)** | ***P*** |
| Order of Procedures |  | | | | | | | | | |
| 1° procedure | Ref. | - | Ref. | - | Ref. | - | Ref. | - | - | - |
| 2° procedure | 0.83  (0.71-0.97) | 0.021 | 0.83  (0.70-0.97) | 0.023 | 0.81  (0.63-1.05) | 0.12 | 0.77  (0.52-1.13) | 0.2 | - | - |
| 3° procedure | 0.71  (0.56-0.89) | 0.004 | 0.76  (0.60-0.96) | 0.022 | 0.73  (0.49-1.08) | 0.12 | 0.36  (0.13-0.99) | 0.047 | - | - |
| 4° procedure | 0.76  (0.57-1.00) | 0.052 | 0.76  (0.57-1.02) | 0.064 | 0.53  (0.30-0.94) | 0.030 | 0.17  (0.02-1.19) | 0.074 | - | - |

*^f^* Odds ratios were adjusted for the patient preoperative risk, surgeon’s age, and surgeon’s faculty status

* Results regarding inpatient death are not provided due to the non-convergence of the model because of the insufficient number of events

**eTable 7. Adjusted Odds Ratios (aOR) for each outcome based on Surgeon Cumulative Operative Time on the Same Day Prior to the Operation (binary exposure) in Surgical Cases Performed Before or After Noon *^f^***

|  | Composite Adverse Events | | Major Surgical Complications | | Unplanned Reoperation | | Extended Stay in Intensive Care Unit | | Death | |
| --- | --- | --- | --- | --- | --- | --- | --- | --- | --- | --- |
|  | **aOR**  **(95% CI)** | ***P*** | **aOR**  **(95% CI)** | ***P*** | **aOR**  **(95% CI)** | ***P*** | **aOR**  **(95% CI)** | ***P*** | **aOR**  **(95% CI)** | ***P*** |
| Cumulative Operative Time spent the Same Day until Operation by Duration of Surgery by Start of First procedure Incision (before/after noon) | | | | | | | | | | |
| 0 min | Ref. | - | Ref. | - | Ref. | - | Ref. | - | Ref. | - |
| >0 min (before noon) | 0.81  (0.70-0.94) | 0.005 | 0.82  (0.71-0.95) | 0.009 | 0.76  (0.60-0.97) | 0.03 | 0.67  (0.45-0.98) | 0.04 | 0.82  (0.40-1.70) | 0.6 |
| >0 min (after noon) | 0.98  (0.64-1.51) | >0.9 | 1.10 (0.70-1.72) | 0.7 | 0.82  (0.38-1.73) | 0.6 | 0.86  (0.28-2.68) | 0.8 | - | - |

*^f^* Odds ratios were adjusted for the patient preoperative risk, surgeon’s age, and surgeon’s faculty status

**eFigure 1. Unadjusted Comparison of Surgical Outcomes based on Surgeon Cumulative Operative Time on the Same Day Prior to the Operation grouped in four categories**
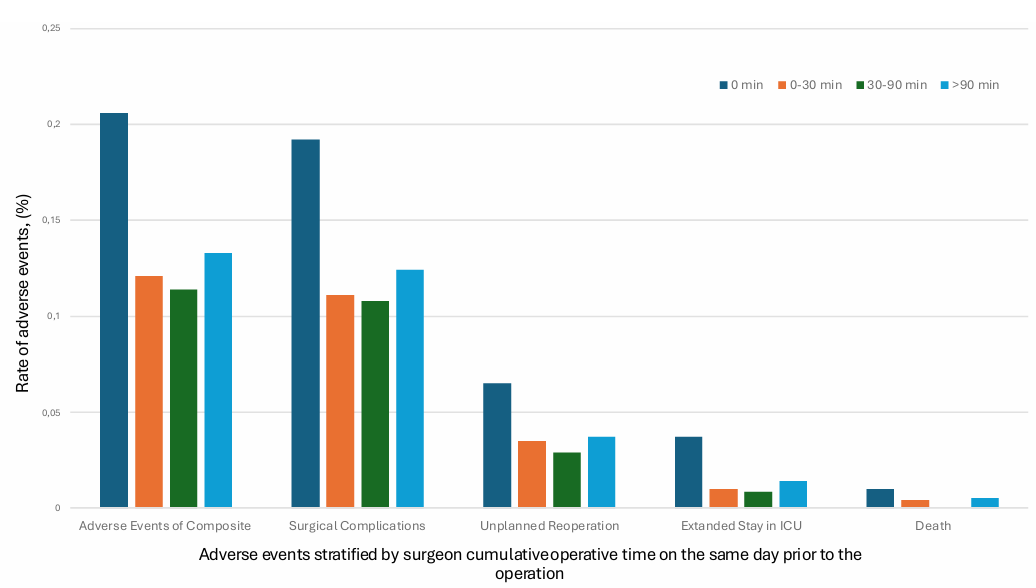


**eFigure 2. Forest Plot overall elective population**


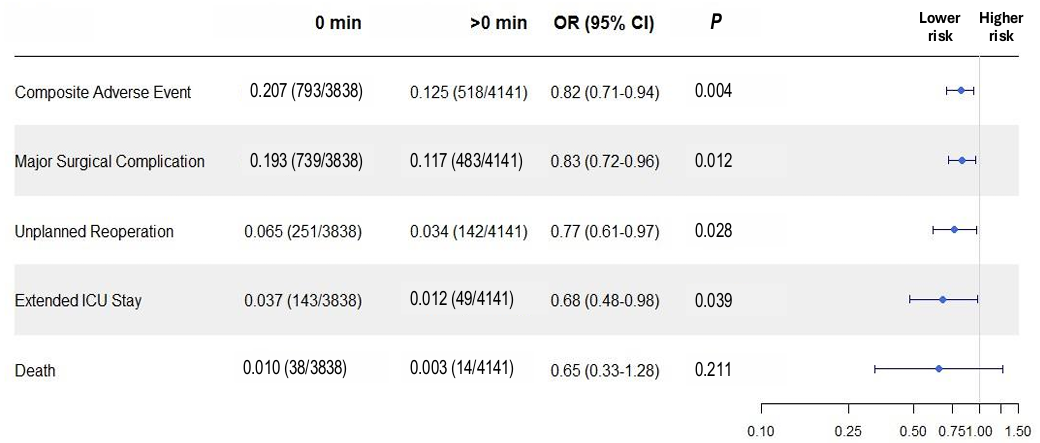


**eFigure 3. Forest Plot elective population stratified by specialty groups**


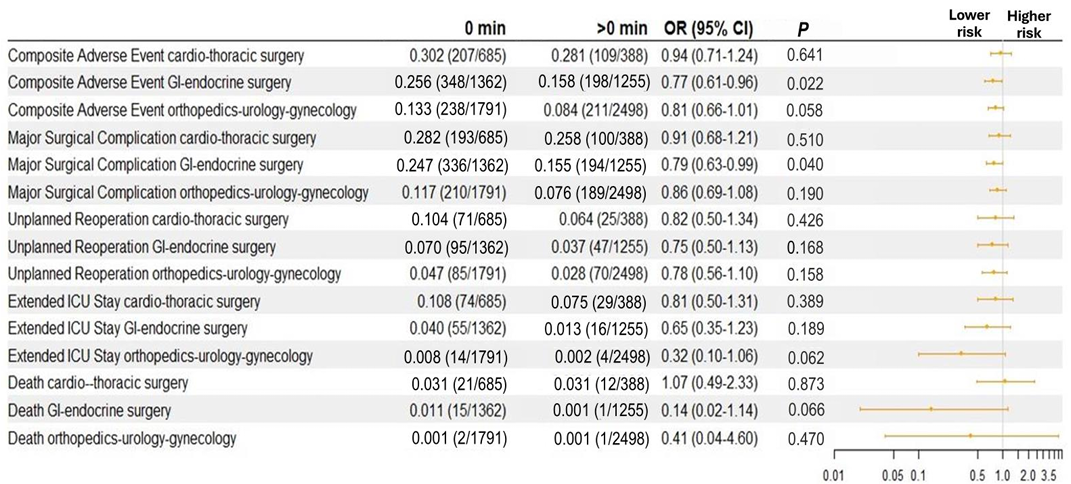


**Methods S1. Patient’s preoperative risk score development**

Surgical outcome was a composite morbidity-mortality assessment criterion including, in the operating room during the initial surgery or within 30 days following the initial surgery, the detection of at least one of the following major adverse events during the period of hospitalization when the procedure was performed or during a subsequent hospitalization:

- Severe complication:
  - Intraoperative complication that occurred during the initial surgery between the time of entry and exit from the operating room: multi-organ failure, shock (hemorrhagic, cardiogenic, septic, or anaphylactic), cardiac arrest/ myocardial infarction, major hemorrhage, hemodynamic instability, accidental wound, material oversight, procedure error, or surgical conversion;
  - Postoperative complication that occurred after discharge from the operating room on the same day or within 30 days following the initial surgery: general (multi-organ failure, implantable medical device or graft failure), infectious (sepsis, organ or surgical site infection, deep infection, implantable medical device or graft infection, pulmonary infection), hemorrhagic (hemorrhage, hemorrhagic shock, deep hematoma, hemarthrosis), parietal (eventration or evisceration), cardiopulmonary (cardiac arrest, myocardial infarction, acute heart failure, acute respiratory failure), neurological (coma, stroke), abdominopelvic (anastomotic complication, fistula, ascites, acute liver failure, intestinal obstruction, digestive necrosis, acute renal failure, pyeloureteral obstruction), orthopedic (fracture, dislocation, ischemia, compartment syndrome, paralysis), cervical (recurrent paralysis, hypoparathyroidism, suffocating hematoma), functional (undernourishment, transit stop);
- Unplanned reoperation for complications related to the initial surgery, including any return of the patient to the operating room for a new surgical procedure with open, video, endoscopic, or interventional radiology approach;
- Postoperative transfer in intensive or intermediate care unit related to organ failure during or after the initial surgery with extended stay (of at least two nights in intensive care unit, at least five nights in intermediate care unit) or ended by death;
- Intraoperative/postoperative death, regardless of the cause.

A preoperative risk score was specifically developed for each of the five outcomes (composite, intraoperative/postoperative complication, unplanned reoperation, extended intensive or intermediate care unit stay, and death) across seven different surgical specialties (cardiac, digestive, endocrine, gynecology, orthopedic, urology, and thoracic surgery). Each risk score model was designed using an independent train dataset (a 50% random sample) of 3,644 operations performed by the same cohort of surgeons at a different period, from January 1st, 2022, to October 31st, 2022. The following variables were systematically considered for inclusion in the models:

- Surgical procedure (767 distinct types of procedures spanning various surgical specialties)
- Surgical indication of the operation based on the chapter of the ICD-10 codes
- Scheduling of the operation (urgent, semi-urgent, elective)
- Type of anesthesia (general, regional, local) and surgical approach (open, videoscopic, endoscopic, robot)
- Patient demographics (age, sex) and socio-economic status (median income of the municipality of residence in quartiles and precarious situation)
- ASA physical status classification system (from 1 to 5)
- Comorbidities including critical condition, current pregnancy, obesity (BMI ≥ 30kg / m²), malnourishment, tobacco addiction, alcohol addiction, other addiction, open wound, surgical site infection, sepsis, endocarditis, cancer, neoadjuvant treatment, immune deficiency, coagulopathy, anticoagulant treatment, anti-aggregation treatment, blood transfusion, coma, limb paralysis, other neurological disorder, confusion, dementia, depression, cardiovascular disease, neurovascular disease, peripheral arterial disease, cardiac arrhythmia, chronic heart failure, hypertension, diabetes, dyslipidemia, pulmonary artery systolic pressure (> 60 mmHg), chronic renal failure, acute renal failure, chronic respiratory failure, chronic obstructive pulmonary disease, liver disease, rheumatic pathology, and hypoparathyroidism.

Considered all those potential confounders, models were subsequently trained using the operations from the independent dataset for each outcome and specialty, occasionally grouping certain specialties due to low event rates, as follows: cardiac with thoracic surgery, digestive with endocrine surgery, and orthopedic with urologic and gynecologic surgery. Given the infrequent occurrences of extended stays in critical care and death, we grouped all specialties together for these outcomes and included the specialty as a covariate in the model. Variables were systematically selected using an automated stepwise logistic regression approach (entry threshold p = 0.20, exit threshold p = 0.10), enabling us to retain a specific set of variables for each specialty and outcome group. The obtained beta coefficients were then applied to the operations of the present study (a 100% exhaustive sample of procedures performed by the cohort of surgeons, from November 1, 2020, to December 31, 2021), allowing us to estimate the probability of adverse event occurrence for each operation during the observation period.

Models performance was evaluated based on their calibration and discrimination with C-statistics and corresponding AUC on the implementation train dataset and on the observation test dataset for accuracy (external validation). The overall discrimination of models in terms of c-statistics and accuracy was 0.854 and 82.2% for the composite, 0.850 and 83.3% for severe complications, 0.858 and 93.2% for reoperations, 0.907 and 99.4% for extended stays in critical care, and 0.955 and 99.5% for deaths.

Below are the performance for the risk score models developed for each outcome.

| Surgical Outcome | Accuracy | Calibration | Discrimination |
| --- | --- | --- | --- |
| Composite | Train : 86.5%  Test : 82.2% | 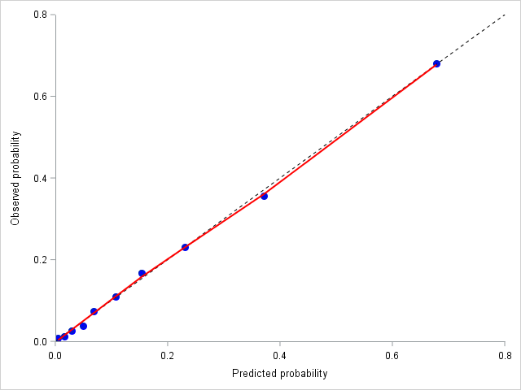 | 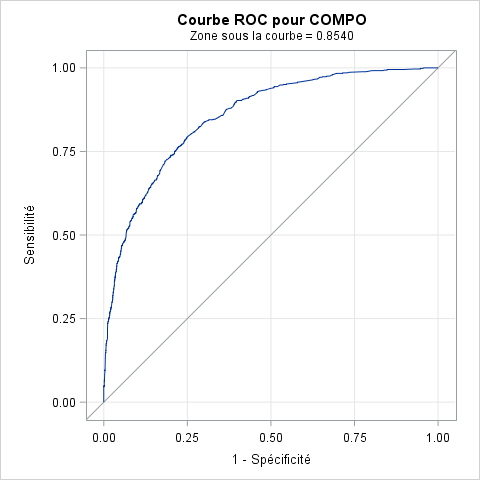  C-Stat=0.854 |
| Severe complications | Train : 87.0%  Test : 83.3% | 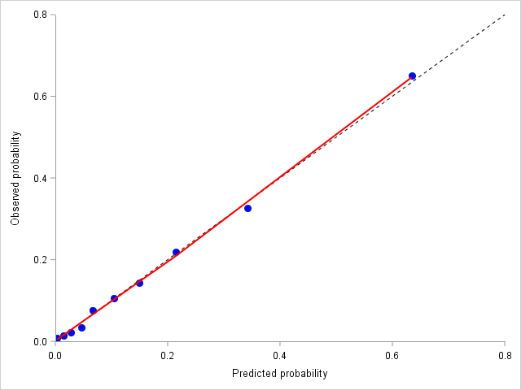 | 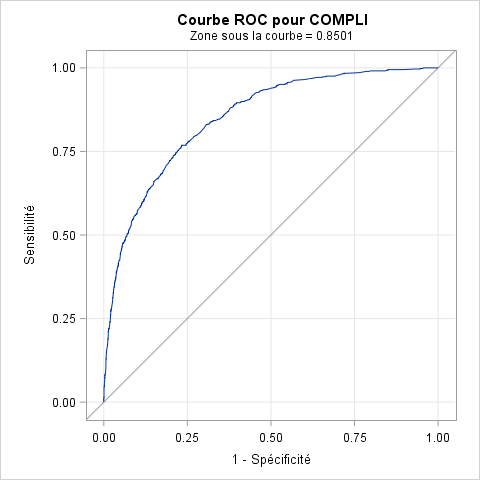  C-Stat=0.850 |
| Unplanned reoperation | Train : 94.0%  Test : 93.2% | 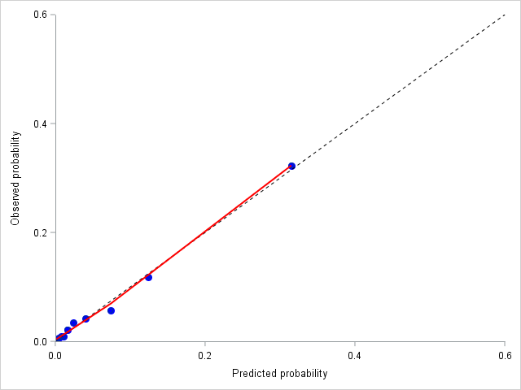 | 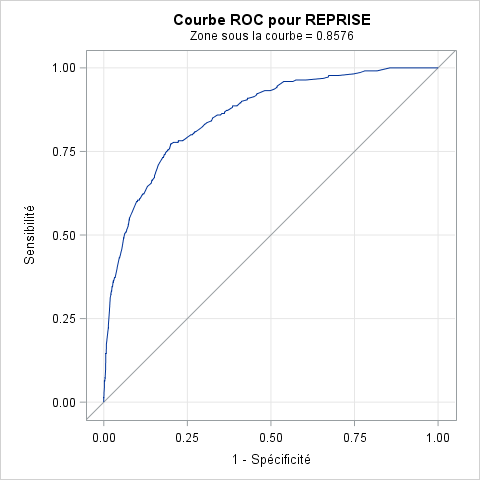 C-Stat=0.858 |
| Extended intensive or intermediate care unit stay | Train : 96.3%  Test : 99.4% | 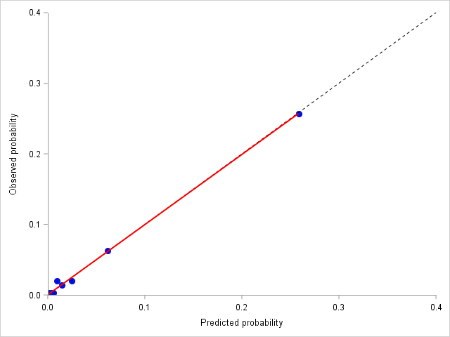 | 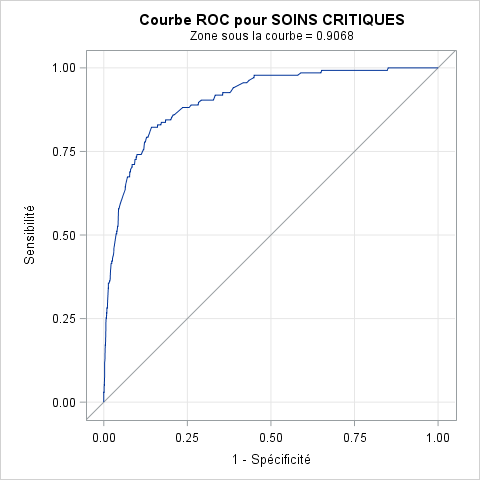  C-Stat=0.907 |
| Death | Train : 98.9%  Test : 99.5% | 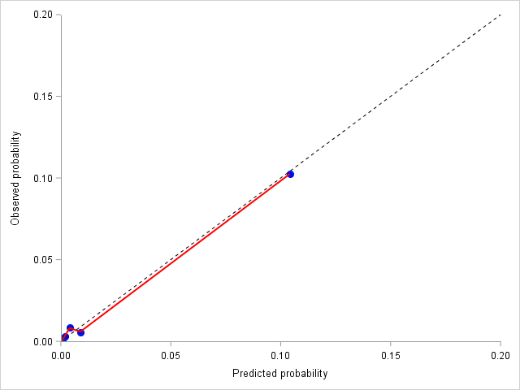 | 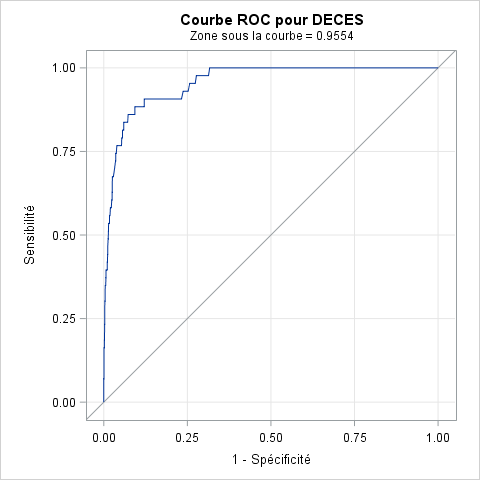  C-Stat=0.955 |
